# A radiomic nomogram based on arterial phase of CT for differential diagnosis of ovarian cancer

**DOI:** 10.1007/s00261-021-03120-w

**Published:** 2021-06-04

**Authors:** Yumin Hu, Qiaoyou Weng, Haihong Xia, Tao Chen, Chunli Kong, Weiyue Chen, Peipei Pang, Min Xu, Chenying Lu, Jiansong Ji

**Affiliations:** 1 Key Laboratory of Imaging Diagnosis and Minimally Invasive Intervention Research, Lishui Hospital of Zhejiang University, Lishui, 323000 China; 2 GE Healthcare, Hangzhou, 310000 China

**Keywords:** Primary ovarian cancer, Secondary ovarian cancer, Radiomics, Nomogram, Differential diagnosis

## Abstract

**Purpose:**

To develop and validate a radiomic nomogram based on arterial phase of CT to discriminate the primary ovarian cancers (POCs) and secondary ovarian cancers (SOCs).

**Methods:**

A total of 110 ovarian cancer patients in our hospital were reviewed from January 2010 to December 2018. Radiomic features based on the arterial phase of CT were extracted by Artificial Intelligence Kit software (A.K. software). The least absolute shrinkage and selection operation regression (LASSO) was employed to select features and construct the radiomics score (Rad-score) for further radiomics signature calculation. Multivariable logistic regression analysis was used to develop the predicting model. The predictive nomogram model was composed of rad-score and clinical data. Nomogram discrimination and calibration were evaluated.

**Results:**

Two radiomic features were selected to build the radiomics signature. The radiomics nomogram that incorporated 2 radiomics signature and 2 clinical factors (CA125 and CEA) showed good discrimination in training cohort (AUC 0.854), yielding the sensitivity of 78.8% and specificity of 90.7%, which outperformed the prediction model based on radiomics signature or clinical data alone. A visualized differential nomogram based on the radiomic score, CEA, and CA125 level was established. The calibration curve demonstrated the clinical usefulness of the proposed nomogram.

**Conclusion:**

The presented nomogram, which incorporated radiomic features of arterial phase of CT with clinical features, could be useful for differentiating the primary and secondary ovarian cancers.

## Introduction

Ovarian cancer is the fifth leading cause of cancer deaths in women in the USA; nearly 300,000 new ovarian cancer patients and 190,000 deaths were found worldwide [1], with five-year survival rate around 40% [2]. Secondary cancers of the ovary account for 10–25% of all ovarian malignancies. The most common cancers that give rise to ovarian metastases include breast, colorectal, endometrial, stomach, and appendix cancer [3]. The primary ovarian cancer (POC) shares similar morphological features with secondary ovarian cancer (SOC), however making it difficult to distinguish on diagnostic imaging [4, 5]. Once identified as SOC, the treatment strategies will be greatly changed [6]. Therefore, differentiating SOC from POC is critical for the identification of precise, personalized treatment, and follow-up plans that prolong patient survival.

Contrast-enhanced computed tomography (CT) is a routinely used tool for diagnosing ovarian cancer non-invasively [7]. Radiomics is an emerging translational field of research aiming to extract mineable high-dimensional data from clinical images, which was followed by subsequent data analysis for decision support [8–10]. It has been successfully employed in the research of oncology, especially for differentiating the primary and metastatic cancer [11, 12]. Because the difference in morphology between POC and SOC may be reflected in CT invisibly, we hypothesized that, by applying radiomics, we could extract and quantify the difference in CT images between POC and SOC.

In this retrospective study, we aimed to evaluate the feasibility of radiomic analysis on contrast-enhanced CT imaging in identifying computer-extracted texture differences between POC and SOC that may not be visually appreciable on conventional CT.

## Methods

### Patients

**Fig. 1:**
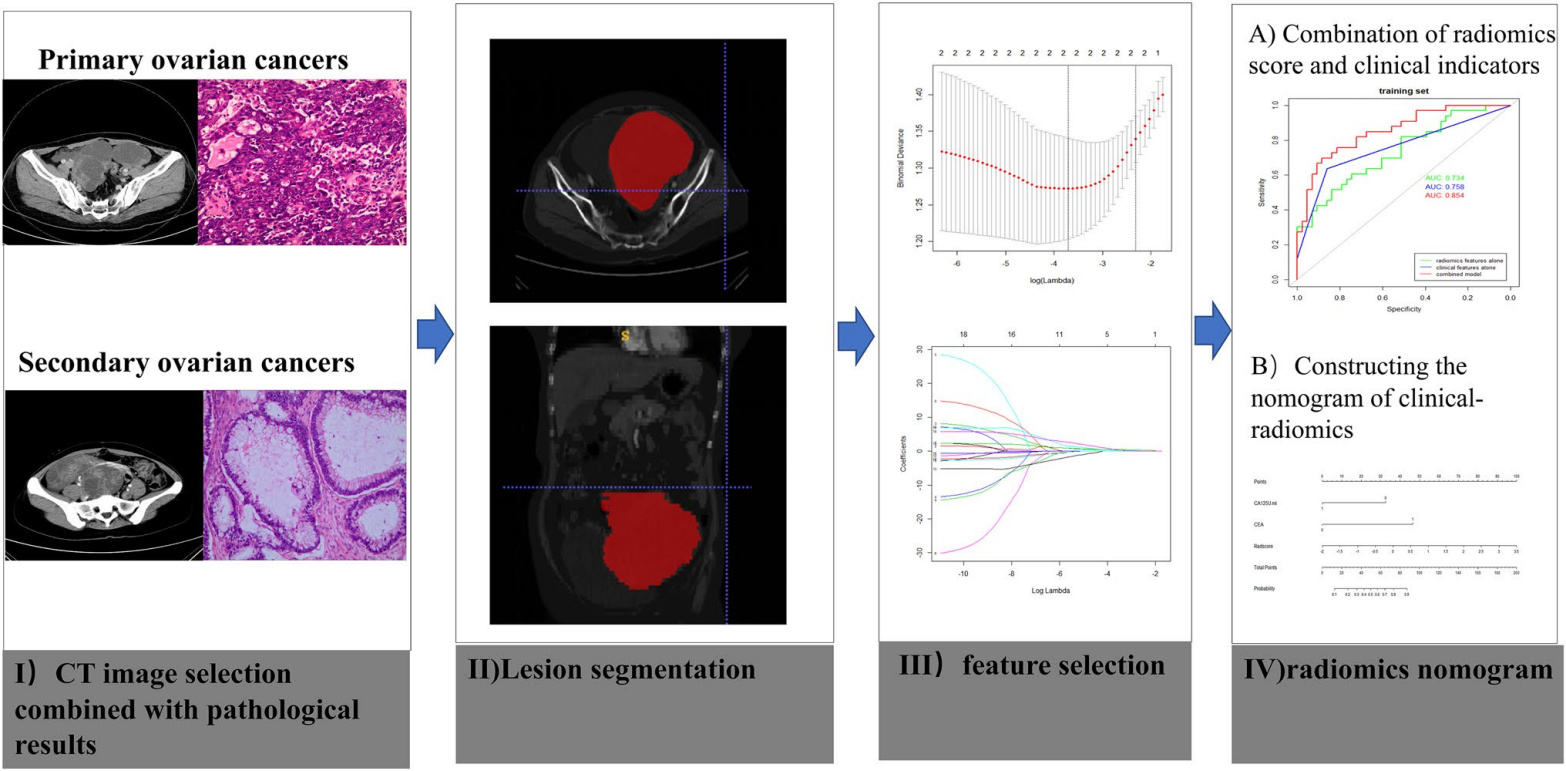
The CT radiomics analysis process from extraction to model building. Workflow can be divided into four steps: image acquisition, lesion segmentation, feature selection, and model construction

**Fig. 2:**
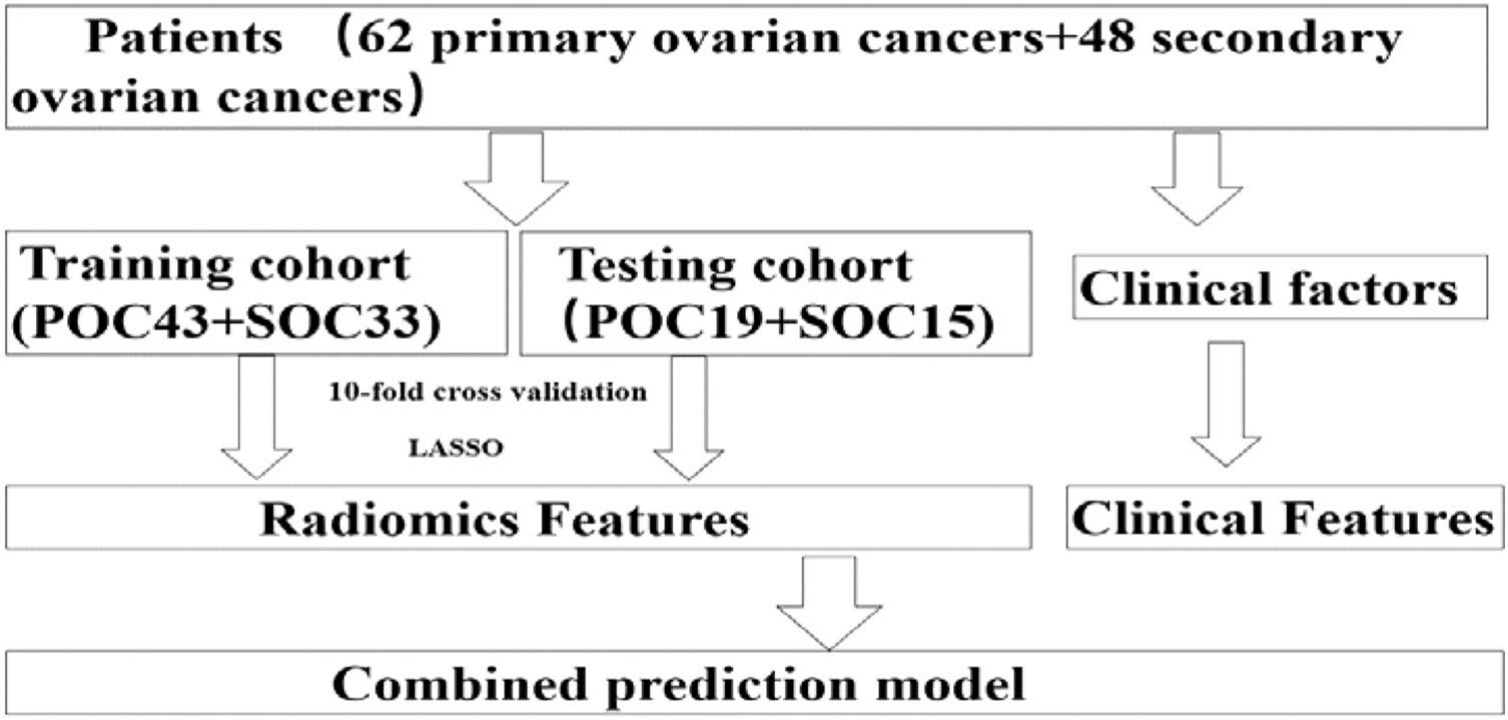
The flow chart of patient selection

Our institutional review board approved this retrospective study with a waiver of informed consent. We reviewed electric database of POC and SOC from January 2010 to December 2018 and retrieved the images of enrolled patients from the picture archiving and communication system (PACS) system. The exclusion criteria of this study are as follows: (1) Patients with poor image quality; (2) patients without enhanced scanning; (3) patients with unclear boundary and unable to outline. Eligible patients including 62 POC patients and 48 SOC patients were randomly divided into the training cohort and the validation cohort. We collected 48 SOC patients, including 20 metastasis of gastric cancer, 15 metastasis of colorectal cancer, and 13 metastasis of sigmoid colon cancer. Among the 48 SOC patients, 18 patients were found to have ovarian masses before the primary malignancy revealed. The post-processing process of the image is shown in Fig. 1, and the flow chart of the whole study is shown in Fig. 2.

### CT image acquisition

Preoperative abdominal CT scans were obtained using a Brilliance ICT (Philips Medical Systems, the Netherlands). The scanning protocol includes unenhanced and contrast-enhanced CT with arterial phase after intravenous administration of iodine-contrast agent (320 mg I/mL) at a rate of 3.0 mL/s with a dose of 2 mL/kg using an automated power injector. The scan parameters were tube voltage of 120 kVp, a pitch value of 0.99, a matrix of 512 × 512, slice thickness and interval both of 5 mm, and milliamperage was adjusted automatically according to the patient's size (ranged between 220 and 400 mA).

### Lesion segmentation and feature selection

The contrast-enhanced CT images of enrolled patients were exported in Digital Imaging and Communication in Medicine (DICOM) format. Then ITK-SNAP was employed for tumor segmentation. We used the polygon mode to delineate cystic and solid component of tumors at each slice manually. All segmentations were conducted by a radiologist (C.L.) who had 16 years of imaging diagnosis experience and re-checked by H.Z. (15 years of experience).

Radiomics features including Histogram, Formfactor, Grey-level size zone matrix(GLSZM), and Run-length matrix (RLM) were extracted by AK software (Artificial Intelligence Kit V3.0.0. R, GE Healthcare, China) [13]. The preprocessing before feature selection was divided into three steps. Firstly, we sought to identify the features that contribute to the model using the ANOVA + KW test. And then we used the binary logistic regression analysis to rule out features, in which the correlation coefficient was greater than 0.9. Finally, the LASSO Cox logistic regression model was used to select the most useful prediction features. Then, the radiomics score (Rad-score) was computed for each patient through a linear combination of selected features weighted by their respective coefficients [14].

### Development of individualized radiomics nomogram

Multivariate logistic regression analysis was used to evaluate the significant clinical factors for distinguishing POC and SOC. Radiomics signature was applied to develop a distinguish model by using the training cohort. Then we combine the rad-score with clinical indicators and established a combination model. The normal reference range for CEA values is 0–10U/ML, for CA125 value is 0–35U/ML, and for CA199 value is 0–37U/ML. Finally, we constructed a visualized nomogram based on the combination model. The calibration curve was used to assess the nomogram. The Hosmer–Lemeshow test was performed to evaluate the goodness-of-fit of the nomogram.

### Statistical analysis

Continuous and categorical variables were compared using the *t* test and chi-square test, respectively. Multivariable logistic regression analysis was used to select the independent prognostic factors. The performance of the model was assessed in the primary and validation cohorts. The discrimination of the signature was measured by the area under the curve (AUC). Statistical analysis was performed with SPSS (version 19.0, IBM, Armonk, NY, USA). A two-sided p value was always computed, and a difference was considered significant at *P* < 0.05.

## Results

### Characteristics of patients in the training and the validation cohorts

A total of 110 ovarian cancer patients were eligible for this study. The 110 patients were divided into a training cohort (*N* = 76) and a testing cohort (*N* = 34) (Fig. 2). The baseline of enrolled patients in training and validation cohorts is displayed in Table 1. There were 56.6% (43/76) of POC patients in the training cohort and 55.9% (19/34) in the validation cohort. The mean age of the patients in the training cohort was 54.74 ± 11.01 years for patients with POC and 50.79 ± 10.24 years with SOC (*P* = 0.114). Significance differences between POC and SOC were found in CEA and CA125 level in the training and validation cohort (all *P* < 0.05).

**Table 1:** Baseline of patients in training and validation cohorts

Characteristics	Training cohort (*n* = 76)			Validation cohort (*n* = 34)		
	POC (*n* = 43)	SOC (*n* = 33)	*P* value	POC (*n* = 19)	SOC (*n* = 15)	*P* value
Age, mean ± SD	54.74 ± 11.01	50.79 ± 10.24	0.114	58.21 ± 8.92	53.53 ± 13.01	0.223
CEA level (n)			0.031			0.047
Normal	41	25		17	9	
Abnormal	2	8		2	6	
CA125 level (n)			0.000			0.019
Normal	5	26		2	7	
Abnormal	38	7		17	8	
CA199 level (n)						
Normal	38	25	0.914	14	7	0.113
Abnormal	5	8		5	8	
Radiomics score(mean ± SD)	− 1.08 ± 1.26	2.65 ± 7.54	0.002	− 0.64 ± 0.87	0.311 ± 1.28	0.015

### Radiomics signature development

**Fig. 3:**
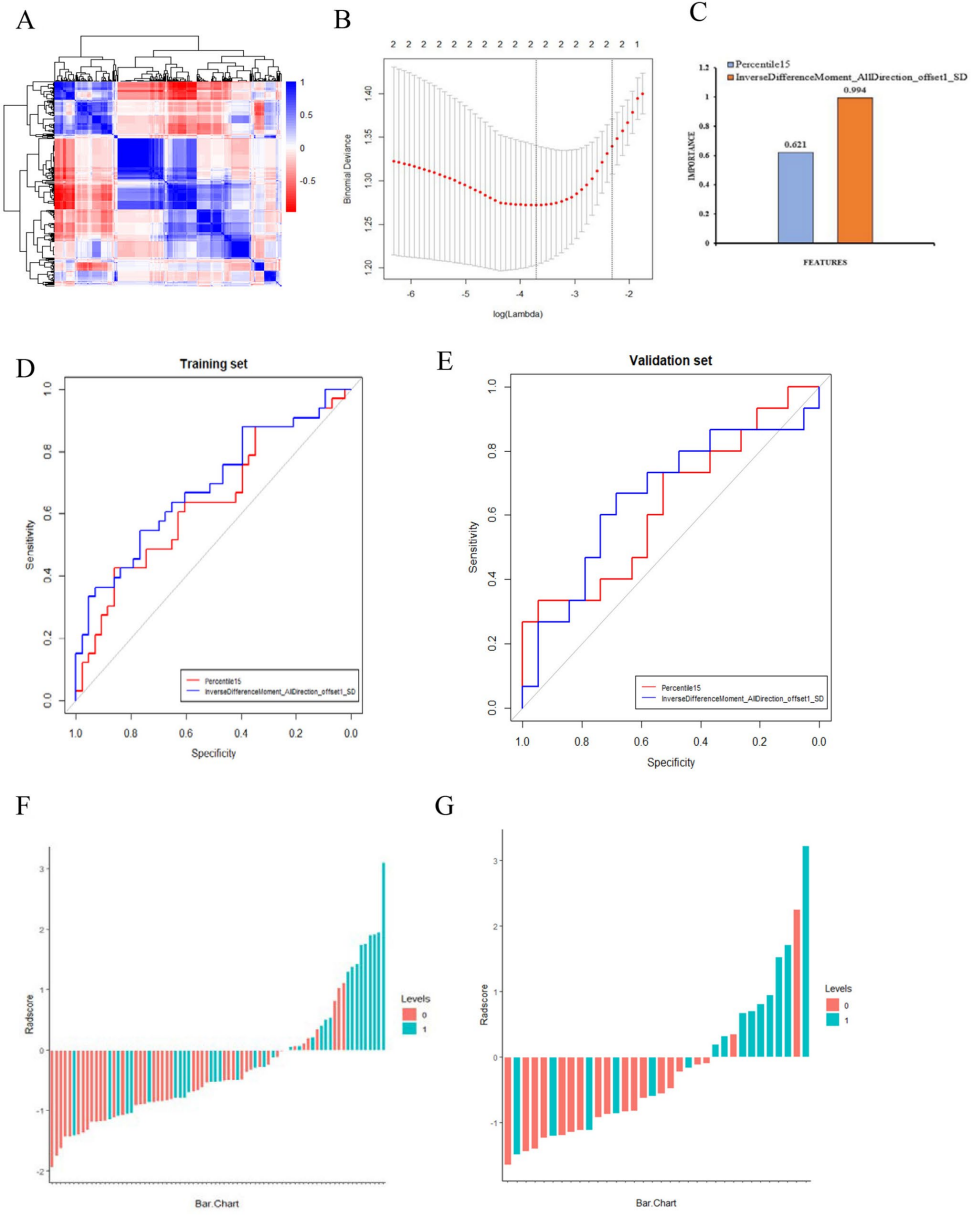
**a** Graph shows correlation analysis between the parameters of training data. **b** Tuning parameters (λ) selected in the LASSO model applied tenfold cross-validation via the minimum criteria. The Y-axis indicates the binomial deviances. The lower X-axis indicates the log(λ). **c** Histogram showing the contribution of each feature to the radiomic signature. **d**–**e** ROC curves of the radiomic signature in the training and validation cohorts. **f**–**g** The two figures showed that the rad-score for patients in training and validation cohort. Red bars represent the scores for POC patients, while blue bars represent the scores for SOC patients

A total of 396 radiomic features were extracted by AK software. After dimensionality reduction, which included ANOVA and KW, univariate logistic regression (14 features) removes the redundancy with correlation coefficient more than 0.90 (5 features) (Fig. 3a) and after the LASSO algorithm with λ value of 0.0245 and log (λ) value of 3.71, two significant radiomic features were identified [15, 16] (Fig. 3b). Two potential radiomic features including Percentile15 and Inverse Difference Moment_AllDirection_offset1_SD remained after dimension reduction with LASSO. The contribution of the selected features and their corresponding regression coefficients are shown in Fig. 3c. The two characteristics of the ROC curve of training and validation are shown in Fig. 3d–e.These features were presented in the rad-score calculated by using the following formula:

rad-score =  − 0.220 + 0.621*Percentile15 + 0.994*InverseDifferenceMoment_AllDirection_offset1_SD.

The Rad-score for each patient presented as a waterfall plot demonstrated significant differences between POC and SOC in both training (*P* = 0.001) and testing cohorts (*P* = 0.015) (Fig. 3f–g). The radiomics signature also showed a favorable predictive efficacy, with an AUC of 0.734 in the training cohort (95% CI 0.620–0.847, sensitivity = 51.5%, specificity = 83.7%) and 0.733 in the validation cohort (95% CI 0.549–0.917, sensitivity = 60%, specificity = 89.5%).

### Nomogram building and validation

**Fig. 4:**
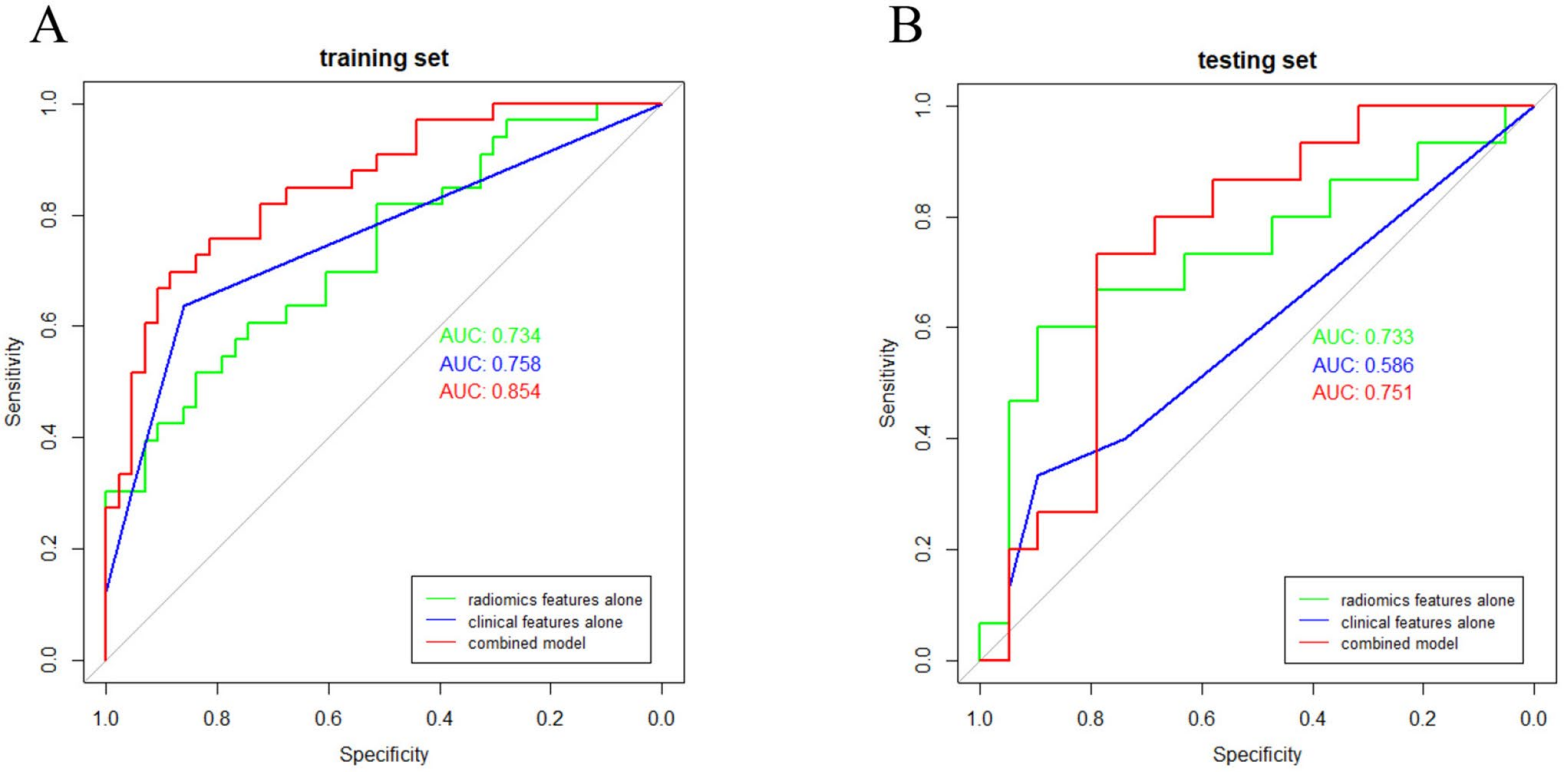
Receiver operating characteristic (ROC) curve of clinical features model, radiomic features model, and combination model in training cohort (**a**) and testing cohort (**b**)

**Fig. 5:**
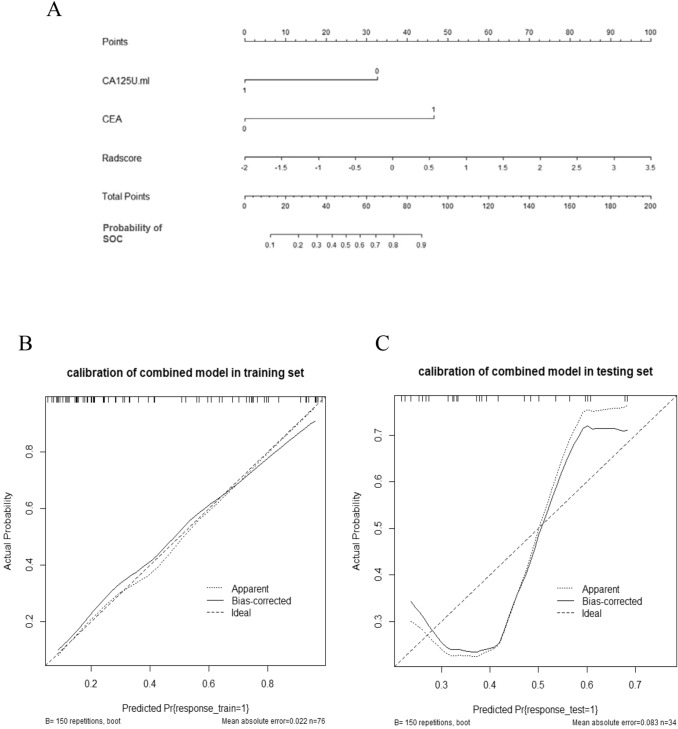
Constructed multiparametric radiomics nomogram and calibration curves. **a** The developed radiomic nomogram for differentiating POC and SOC. **b**–**c** Calibration curves for differentiating POC and SOC in the training and validation cohort, respectively. The calibration curve illustrates the calibration of the nomogram in terms of the agreement between the predicted risk of SOC and the observed outcomes. The diagonal dotted line represents a perfect prediction, and the dotted line represents the predictive performance of the nomogram. Closer fit to the diagonal dotted line indicates a better prediction

The rad-score, CEA, and CA125 were identifed as independent factors to distinguish POC and SOC by logistic regression. Then, we integrated the above factors into a prediction model. As shown in Fig. 4a–b, the combination model outperformed the radiomic features only model and clinical features only model with a greater AUC of 0.854 in training cohort and 0.751 in testing cohort. Finally, a radiomic-based nomogram for individualized differentiation was built by corporating the above radiomic features and 2 clinical factors (Fig. 5a). The total points accumulated by various variables correspond to the predicted probability for a patient. The calibration curve of the radiomics nomogram demonstrated good agreement in both training and validation cohorts. (Fig. 5b–c).

## Discussion

Since the POC and SOC representing the different origins and histopathology, differentiating these two sets is especially crucial due to their rather different treatments and prognoses [10, 17]. POC shares overlapping morphological with SOC, making the differential diagnosis rather difficult by conventional imaging modalities. In the present retrospective study, we developed and validated a diagnostic model, radiomics signature–based nomogram in a training cohort for the individualized distinguish the POC and SOC. The nomogram incorporates two items of the radiomics signature, CEA status, and CA125 status. To the best of our knowledge, this is the first study to develop a radiomics-based nomogram to distinguish POC and SOC.

Radiomics is designed to develop decision support tools; therefore, it involves combining radiomic data with other patient characteristics, as available, to increase the performance of the decision support models [18–20]. With the growth of clinical data and advanced machine-learning methodologies, it is playing an increasingly important role in precision diagnostics and oncology. Besides, radiomics has the capability to mine differentiation information from CT [21, 22].

Serum CA-125 is elevated in about 80% of advanced ovarian cancers, which has been considered as a specific marker for the diagnosis of ovarian cancer cells [23]. CEA is a classic broad-spectrum tumor marker mainly used in the diagnosis of digestive tract cancer and lung cancer. CEA levels are elevated in some patients with ovarian cancer [24]. However, serum CA-125 levels and CEA levels do not provide any information about the locations or extent of cancers [25]. Therefore, combining radiomic features with clinical features could provide added diagnostic value in identifying POC and SOC. This was first confirmed in our article that the AUC of combination model was much higher than that of clinical features or radiomic features model.

This study has several limitations. First, owing to the insufficient sample size, the differentiation performance may be limited. Further studies are required to include more patients from multi-institution to fully assess the generalization ability of the radiomics model in future. Second, as the nature of retrospective study, selection bias would be existed; thus, a prospective external validation is required. Third, manual segmentation for ROI is time-consuming and may not completely avoid the interference caused by the partial volume effect.

## Conclusion

In summary, by comparing the clinical features model, radiomic features model, and combination model, we found that the combination model achieved the best diagnostic performance. Therefore, we believe that the combination model, which integrated clinical and radiomic features, could be used as non-invasive and reliable tool for differentiating POC and SOC pre-treatment. In addition, we successfully developed and validated a convenient prediction nomogram that can be used to identify SOC patients.

## Data Availability

The data sets used and/or analyzed during the current study available from the corresponding author on reasonable request.

## Abbreviations

POC: Primary ovarian cancer
SOC: Secondary ovarian cancer
LASSO: Least absolute shrinkage and selection operation regression
CA125: Carbohydrate antigen 125
CEA: Carcino-embryonic antigen
PACS: Picture archiving and communication systems
DCA: Decision curve analysis
GLSZM: Grey-level size zone matrix
RLM: Run-length matrix
ANOVA: Analysis of variance
KW: Kruskal–Wallis
